# Supplementary material for: Linkage-aware inference of fitness from short-read time-series genomic data
Source: Virus Evol. 2026 Apr 25;12(1):veag027. doi: 10.1093/ve/veag027 (PMC13191327; doi:10.1093/ve/veag027)
Supplement: veag027_Supplemental_Files [file veag027_supplemental_files.zip › Supplementary_Table_S1_veag027.docx]

| Mut nt  Ref nt | A | C | G | T | - (gap) |
| --- | --- | --- | --- | --- | --- |
| A | 0 | $9.0\times{10}^{-7}$ | $6.0\times{10}^{-6}$ | $7.0\times{10}^{-7}$ | $1.0\times{10}^{-9}$ |
| C | $5.0\times{10}^{-6}$ | 0 | $5.0\times{10}^{-7}$ | $1.2\times{10}^{-5}$ | $1.0\times{10}^{-9}$ |
| G | $1.6\times{10}^{-5}$ | $1.0\times{10}^{-7}$ | 0 | $2.0\times{10}^{-6}$ | $1.0\times{10}^{-9}$ |
| T | $3.0\times{10}^{-6}$ | $1.0\times{10}^{-5}$ | $3.0\times{10}^{-6}$ | 0 | $1.0\times{10}^{-9}$ |
| - (gap) | $1.0\times{10}^{-9}$ | $1.0\times{10}^{-9}$ | $1.0\times{10}^{-9}$ | $1.0\times{10}^{-9}$ | $0$ |

**Table S1. Mutation rates used in the analysis of HIV-1 patient data.** Each entry represents the mutation rate from the nucleotide in the row to the nucleotide in the column.
