# Supplementary material for: Linkage-aware inference of fitness from short-read time-series genomic data
Source: Virus Evol. 2026 Apr 25;12(1):veag027. doi: 10.1093/ve/veag027 (PMC13191327; doi:10.1093/ve/veag027)
Supplement: veag027_Supplemental_Files [file veag027_supplemental_files.zip › Supplementary_Table_S2_veag027.docx]

|  | CD8+ T cell escape mutations | | | Nonsynonymous reversions outside CD8+ T cell epitopes | | | Nonsynonymous reversions within CD8+ T cell epitopes | | |
| --- | --- | --- | --- | --- | --- | --- | --- | --- | --- |
|  | % | Fold enrichment | p-value | % | Fold enrichment | p-value | % | Fold enrichment | p-value |
| MPL-R | $40.5$ | $23$ | $<{10}^{-13}$ | $13.5$ | $21$ | $<{10}^{-5}$ | $10.8$ | $358$ | $<{10}^{-8}$ |
| MPL | $34.3$ | $18$ | $<{10}^{-9}$ | $17.1$ | $27$ | $<{10}^{-7}$ | $11.4$ | $382$ | $<{10}^{-8}$ |
| MPL (iden) | $28.6$ | $14$ | $<{10}^{-7}$ | $22.9$ | $33$ | $<{10}^{-9}$ | $11.4$ | $382$ | ${<10}^{-8}$ |

**Table S2. MPL-R reports similar insights as MPL.** Percentage of mutations, fold enrichment, and p-values reported by MPL-R, MPL, and MPL (identity covariance) are given for mutations in the listed categories in the top 5% mutations with the strongest selection coefficients.
