# Supplementary material for: Linkage-aware inference of fitness from short-read time-series genomic data
Source: Virus Evol. 2026 Apr 25;12(1):veag027. doi: 10.1093/ve/veag027 (PMC13191327; doi:10.1093/ve/veag027)
Supplement: veag027_Supplemental_Files [file veag027_supplemental_files.zip › Supplementary_Table_S3_veag027.docx]

| **Mutation** | **Experimentally reported phenotypic effects** |
| --- | --- |
| S371F | Antibody escape (Cao *et al.*, 2022a, 2022b) |
| F377L | Antibody escape (Huo *et al.*, 2021) |
| D405N | Antibody escape (Cao *et al.*, 2022b) |
| K417N | Antibody escape (Yuan *et al.*, 2021, Wang *et al.*, 2021) |
| N440K | Antibody escape (Rani *et al.*, 2021) |
| N450K | Antibody escape (Liu *et al.*, 2021) |
| Q493R | Antibody escape (Focosi *et al.*, 2021) |
| Q498R | Antibody escape (Cui *et al.*, 2022), host-binding enhancement (Hong *et al.*, 2022) |
| Y505H | Antibody escape (Wang *et al.*, 2023) |
| G339D | T-cell response weakening (Li *et al.*, 2022), antibody escape (Cao *et al.*, 2022a) |
| N354K | Immunogenicity reduction and antibody escape (Liu *et al.*, 2024) |
| L452Q | Stability enhancement (Starr *et al.*, 2022), antibody escape (Li *et al.*, 2020, Cao *et al.*, 2022b), infectivity enhancement (Deng *et al.*, 2021) |
| V367F | Infectivity enhancement (Ou *et al.*, 2021) |
| S375F | Infectivity enhancement (Kimura *et al.*, 2022) |
| S373P | Host-binding enhancement (Zheng *et al.*, 2023) |
| N501Y | Host-binding enhancement (Tian, 2021) |
| T376A | Spike cleavage reduction (Hu *et al.*, 2022) |

Table S3. Mutations inferred as beneficial by MPL-R map to known beneficial mutations. Nonsynonymous mutations in the RBD which are inferred as beneficial by MPL-R and neutral or deleterious by MPL (identity covariance) map to mutations reported to be beneficial to the virus.
